# Collagen fiber features and COL1A1: are they associated with elastic parameters in breast lesions, and can COL1A1 predict axillary lymph node metastasis?

**DOI:** 10.1186/s12885-022-10092-7

**Published:** 2022-09-21

**Authors:** Ying Jiang, Bo Wang, Jun Kang Li, Shi Yu Li, Rui Lan Niu, Nai Qin Fu, Jiao Jiao Zheng, Gang Liu, Zhi Li Wang

**Affiliations:** 1 School of Medicine, Nankai University, 94 Weijin Road, Tianjin, 300071 China; 2 Department of Ultrasound, Chinese People’s Liberation Army General Hospital, 28 Fuxing Road, Beijing, 100853 China; 3 Department of Radiology, Chinese People’s Liberation Army General Hospital, 28 Fuxing Road, Beijing, 100853 China

**Keywords:** SWE, COL1A1, Breast lesions, Lymph node metastasis, Collagen

## Abstract

**Background:**

This study aimed to explore whether collagen fiber features and collagen type I alpha 1 (COL1A1) are related to the stiffness of breast lesions and whether COL1A1 can predict axillary lymph node metastasis (LNM).

**Methods:**

Ninety-four patients with breast lesions were consecutively enrolled in the study. Amongst the 94 lesions, 30 were benign, and 64 were malignant (25 were accompanied by axillary lymph node metastasis). Ultrasound (US) and shear wave elastography (SWE) were performed for each breast lesion before surgery. Sirius red and immunohistochemical staining were used to examine the shape and arrangement of collagen fibers and COL1A1 expression in the included tissue samples. We analyzed the correlation between the staining results and SWE parameters and investigated the effectiveness of COL1A1 expression levels in predicting axillary LNM.

**Results:**

The optimal cut-off values for Emax, Emean, and Eratio for diagnosing the benign and malignant groups, were 58.70 kPa, 52.50 kPa, and 3.05, respectively. The optimal cutoff for predicting axillary LNM were 107.5 kPa, 85.15 kPa, and 3.90, respectively. Herein, the collagen fiber shape and arrangement features in breast lesions were classified into three categories. One-way analysis of variance (ANOVA) showed that Emax, Emean, and Eratio differed between categories 0, 1, and 2 (*P* < 0.05). Meanwhile, elasticity parameters were positively correlated with collagen categories and COL1A1 expression. The COL1A1 expression level > 0.145 was considered the cut-off value, and its efficacy in benign and malignant breast lesions was 0.808, with a sensitivity of 66% and a specificity of 90%. Furthermore, when the COL1A1 expression level > 0.150 was considered the cut-off, its efficacy in predicting axillary LNM was 0.796, with sensitivity and specificity of 96% and 59%, respectively.

**Conclusions:**

The collagen fiber features and expression levels of COL1A1 positively correlated with the elastic parameters of breast lesions. The expression of COL1A1 may help diagnose benign and malignant breast lesions and predict axillary LNM.

## Background

The incidence of female breast cancer in China is increasing annually, seriously affecting the quality of life of women [1]. The axillary lymph nodes are early metastatic sites of breast cancer, and proper preoperative evaluation is key to the formulation of rational treatment regimens. Furthermore, stiffness is an important characteristic of tissues and organs and is the basis for clinicians to diagnose breast abnormalities. The advent of shear wave elastography (SWE) offers the possibility of achieving quantitative judgment of tissue stiffness and can facilitate appropriate degradation of masses with low suspicion of breast imaging reporting and data system (BI-RADS) category 4A at soft borders [2]. Recently, several studies have reported that SWE showed good performance in assessing benign and malignant lesions [3–5]. The stromal reaction that occurs because of invasive behavior results in abnormal tumor-associated collagen deposition, potentially leading to increased elastic modulus values in breast cancer [6]. Previous studies have shown that the elastic parameters of SWE in breast tumors can be used to predict the lymph node status of invasive breast cancer (IBC); however, the pathological basis is unclear [7].

Previous studies on SWE and breast tissue stroma have focused on the relationship between the quantity of collagen or fibrosis and elastic parameters [8]. However, the influence of the shape and arrangement of collagen fibers on the stiffness of the extracellular matrix (ECM) has rarely been considered. Malignant lesions are stiffer than benign lesions because of the presence of desmoplastic ECM, with type I collagen being a major structural component [9]. The primary function of collagen type I is to support organs and tissues and cause tissue to form tension [10]. Additionally, collagen type I is closely related to cell growth, proliferation, differentiation, and inflammation [11]. Moreover, collagen type I consists of a triple-helix structure consisting of α1(I) chains (COL1A1) and α2(I) chains (COL1A2). These two polypeptide chains are encoded by different genes and are generally synthesized at a ratio of 2:1 [12]. Nevertheless, little is known about the functional role of COL1A1 in elastography feature differences and axillary lymph node metastasis (LNM) of breast lesions. This study aimed to explore the relationship between the shape and arrangement of collagen fibers and the elastic parameters of breast lesions, which were evaluated using SWE. Additionally, we evaluated the role of COL1A1 expression in the differential diagnosis of benign and malignant breast lesions and analyzed the feasibility of COL1A1 in predicting axillary LNM.

## Methods

### Patients

From May 2021 to December 2021, 94 patients with breast lesions were consecutively enrolled in this prospective study, and the 94 breast lesions were examined using conventional ultrasound (US) and SWE before surgical resection. The inclusion criterion was hospitalized women with clear pathological diagnosis. In contrast, the exclusion criteria included patients who underwent biopsy or neoadjuvant chemotherapy before US and SWE examinations and patients with pathological multifocal IBC. The pathology results obtained by surgery were regarded as gold criteria. Furthermore, this trial was conducted in accordance with the Declaration of Helsinki (revised in 2013). The study was approved by the Ethics Committee of the Chinese PLA General Hospital (No. S2021-683–01), and informed consent was obtained from all participants. Based on postoperative pathology, the patients were divided into benign and malignant groups. The malignant group was divided into the negative axillary lymph node metastasis (LNM-) group and the positive axillary lymph node metastasis (LNM +) group.

### Preoperative US and SWE examination

SWE was performed after conventional US examination using the AixPlorer US system (SuperSonic Imagine, Aix-en-Provence, France) with an L14-5 linear array probe operating at 4–15 MHz. Furthermore, the same sonographer, with more than 10 years of clinical experience, performed all US and SWE examinations for the breast masses. The probe was applied as lightly as possible to avoid excessive pressure and was maintained as steadily as possible for at least 10–20 s during elastic image acquisition. Additionally, the participants were asked to hold their breath to prevent motion artifacts. Furthermore, to enable a comparison with histopathological data, the maximum imaging section of the lesion was obtained to measure the stiffness of the breast lesion. Conventional US was used to obtain the maximum imaging section of the breast lesion and then start the SWE check, freezing, and measurement. Conversely, attention was paid while adjusting the region of interest (ROI) to include any halo or inelastic abnormal edge lesions while minimizing any normal tissue. The Eratio was obtained by placing the ROI in the normal breast tissue at the same depth as the lesion. This process was repeated three times, and the final values of Emax, Emean, Emin, and Eratio were the average values of these three repetitions.

### Collagen staining and categorization

Sirius red staining was used to assess the shape and arrangement of collagen fibers, and immunohistochemical staining was used to examine the expression of COL1A1. All staining was performed on paraffin sections made from the largest plane of the surgical specimen of the breast tumor. An Olympus BX53P microscope equipped with a polarized filter was used to examine the collagen fibers at 400 × magnification. In this study, the collagen fiber shape and arrangement feature in breast lesions were classified into three categories. The definitions were described according to the following descriptions: category 0, wavy collagen fibers similar to collagen fibers in normal breast tissue; category 1, taut parallel collagen fibers around tumor nests; category 2, straightened and aligned collagen fibers tending to be perpendicular to the tumor boundary [13–16]. Following the classification principle we adopted, five fields of view were randomly selected. Collagen category was determined when the characteristic collagen arrangement exceeded 80% within the five fields. Analysis and classification of collagen fibers in each section were carried out by a physician with five years of experience in histopathology, who was blinded to the SWE and surgical pathology results.

COL1A1 expression was detected by immunohistochemistry (IHC). IHC staining results were analyzed using Image-Pro Plus 6.0 software (Media Cybernetics, Rockville, MD, USA). Five areas of interest were taken from each slice under a 200 × field of view and photographed. After measuring the integrated optical density (IOD) and area, the average optical density (AOD = IOD/Area) was used to evaluate the expression of COL1A1.

### Statistical analysis

All statistical analyses were performed using SPSS (version 25.0; IBM Corp., Armonk, NY, USA). The measurement data are expressed as mean ± standard deviation (SD) and count data as percentages (%). One-way analysis of variance (ANOVA) was used to compare the data between the three groups. The chi-square test was used to compare the rates between the groups. Furthermore, receiver operating characteristic (ROC) curves were constructed for SWE values and COL1A1 expression levels to differentiate benign from malignant breast lesions and predict axillary LNM. The corresponding sensitivity and specificity were recorded (95% confidence interval).

Additionally, the correlation between COL1A1 expression level and elastic parameters in breast lesions was evaluated using Pearson correlation analysis, and the coefficients were defined as follows: < 0.40 indicated poor reliability; 0.40–0.75 indicated good reliability; and > 0.75 indicated excellent reliability [17]. In addition, Kendall's tau-b correlation coefficient was used to determine the correlation between different collagen fiber features and SWE parameters in the 94 cases of breast lesions. A value of *P* < 0.05 was considered statistically significant.

## Results

### Clinicopathological characteristics

**Fig. 1:**
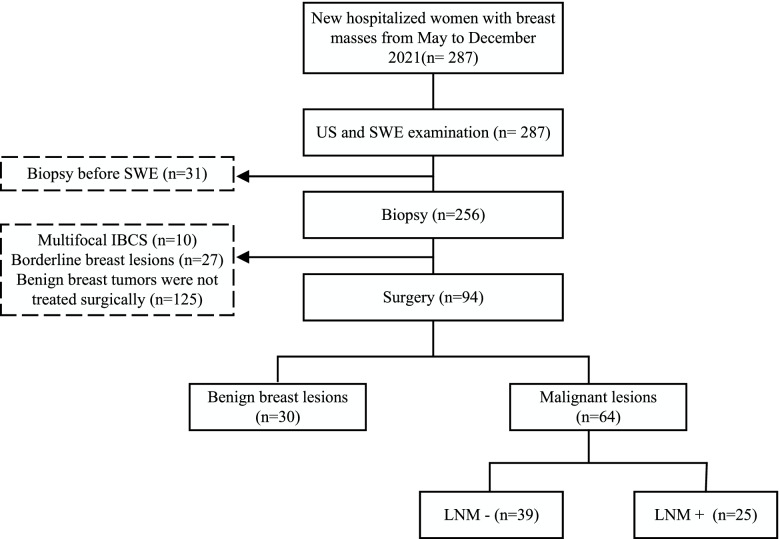
Flowchart of patient enrollment

Overall, 94 breast tumors were surgically removed and histopathologically approved, including 30 benign and 64 malignant lesions (Fig. 1). Benign lesions included fibroadenomas (*n* = 17), sclerosing adenoses (*n* = 5), inflammatory lesions (*n* = 3, including 2 acute suppurative mastitis and 1 granulomatous lobular mastitis), intraductal papillomas (*n* = 4), and benign phyllodes tumor (*n* = 1). The malignant lesions included invasive carcinomas (*n* = 59, including 45 invasive ductal carcinomas, 6 invasive lobular carcinomas, 4 invasive ductal and lobular carcinomas and 4 mucinous carcinomas), and ductal carcinomas in situ (*n* = 5). Furthermore, the presence of axillary lymph nodes was determined based on postoperative pathology, and malignant breast tumors were divided into LNM + and LNM- groups. Amongst the 64 malignant tumors, 25 (39%) were positive for axillary lymphatic metastases, and 39 (61%) were negative (Table 1).

**Table 1:** Clinicopathological information, elasticity parameters, category of collagen fiber, and COL1A1 expression of 94 cases

Parameters	Benign (*n* = 30)	Malignant (*n* = 64) LNM- (*n* = 39) LNM + (*n* = 25)		F/χ2	*P* value
**Age (years)**	44.230 ± 8.947	47.36 ± 8.725	48.24 ± 9.926	1.55	0. 22
**Size (cm)**	2.003 ± 0.615	2.121 ± 0.591	2.408 ± 0.778	2.74	0.07
**Elastic parameters**					
Emax (kPa)	44.450 ± 20.960	90.692 ± 48.697	131.696 ± 57.104	26.33	< 0.001
Emean (kPa)	33.687 ± 18.466	53.331 ± 23.278	91.280 ± 35.102	35.01	< 0.001
Emin (kPa)	20.320 ± 14.422	25.877 ± 19.415	27.068 ± 17.253	1.26	0.29
Eratio	1.927 ± 0.516	3.454 ± 1.594	6.112 ± 2.338	46.79	< 0.001
**Expression of COL1A1**	0.105 ± 0.323	0.140 ± 0.456	0.188 ± 0.332	31.72	< 0.001
**Category of Collagen fiber (n, %)**					
0	25 (83)	10 (25.6)	3 (12)	35.016	< 0.001
1	5 (17)	20 (51.3)	5 (20)		
2	0	9 (23.1)	17 (68)		
**Estrogen receptor (n, %)**	-			0.270	0.603
Positive	-	29 (74.4)	20 (80)		
Negative	-	10 (25.6)	5 (20)		
**Progesterone receptor (n, %)**	-			1.340	0.247
Positive	-	26 (66.7)	20 (80)		
Negative	-	13 (33.3)	5 (20)		
**HER2 status (n, %)**	-			3.770	0.276
0, 1 +	-	13 (33.3)	7 (28)		
2 +	-	18 (46.2)	14 (56)		
3 +	-	8 (20.5)	4 (16)		
**Ki‑67 index (%) (n, %)**	-			6.140	0.013
< 20	-	18 (46.2)	4 (16)		
≥ 20	-	21 (53.8)	21 (84)		

Emax, maximum elasticity; Emean, mean elasticity; Emin, minimum elasticity; Eratio, elasticity ratio of the lesions to the peripheral tissue, *COL1A1* Collagen type I alpha 1, *AOD* Average optical density

### Elastic parameters, collagen fiber features, and COL1A1 expression

**Fig. 2:**
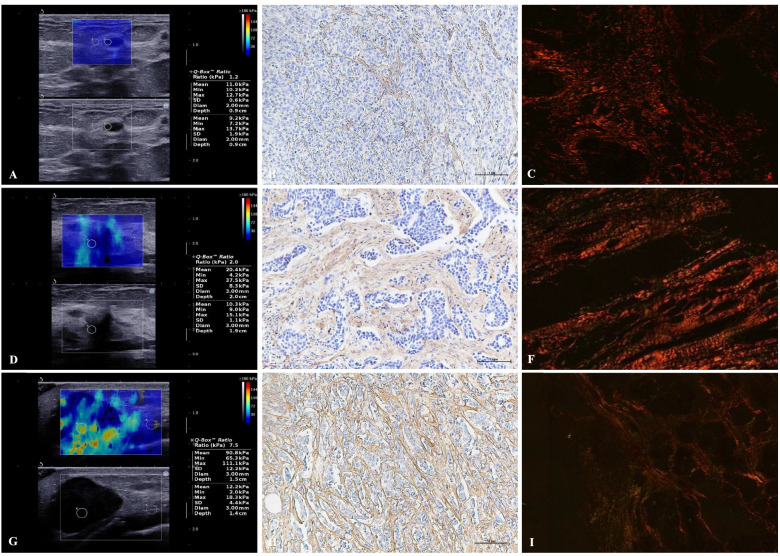
The comparison of SWE (**A**, **D**, **G**), COL1A1 immunohistochemical staining (**B**, **E**, **H**: × 200), and collagen Sirius staining (**C**, **F**, **I**: × 400) of breast lesions between the benign (**A**, **B**, **C**), LNM—(**D**, **E**, **F**) and LNM + (**G**, **H**, **I**) groups. SWE, shear wave elastography; COL1A1, collagen type I alpha 1; LNM, lymph node metastasis

**Fig. 3:**
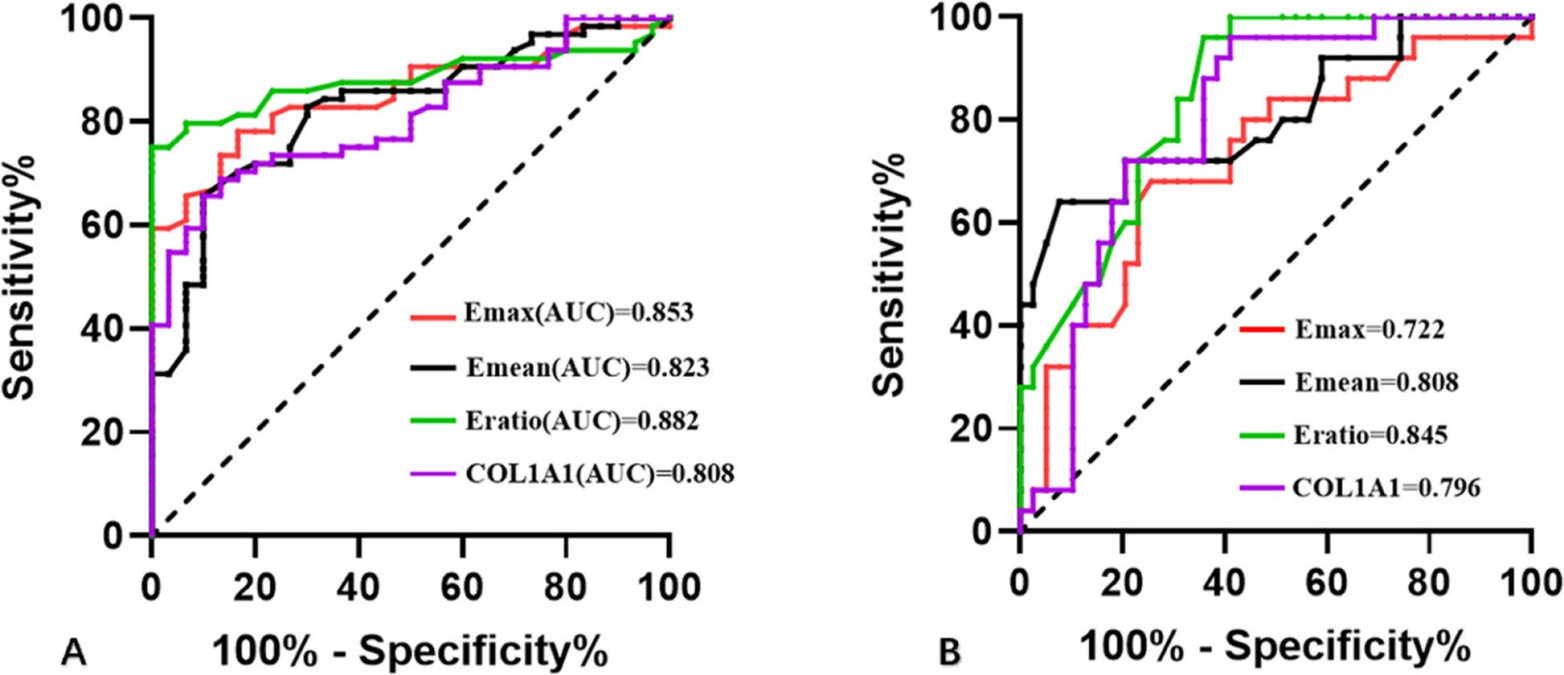
The ROC curve of in the differential diagnosis of three groups. **A** The ROC curve of elastic parameters and the COL1A1 expression level in the differential diagnosis of benign and LNM- groups. **B** The ROC curve of elastic parameters and the COL1A1 expression level in the differential diagnosis of LNM- and LNM + groups. ROC, receiver operating characteristic; COL1A1, collagen type I alpha 1; Emax, maximum elasticity; Emean, mean elasticity; Eratio, elasticity ratio of the lesions to peripheral tissue; LNM, lymph node metastasis

The Ultrasonic elastography, Sirius red staining, and COL1A1 immunohistochemical staining of the included 94 samples are shown in Fig. 2. The ROC curves of Emax, Emean, and Eratio elasticity for the diagnosis of the malignant and benign groups are shown in Fig. 3. Furthermore, the optimal cut-off values yielding the maximum sensitivity and specificity were greater than 58.70 kPa, 52.50 kPa, and 3.05. The areas under the ROC curves were 0.853 (0.779–0.928), 0.823 (0.737–0.910), and 0.882 (0.813–0.951), respectively.

**Fig. 4:**
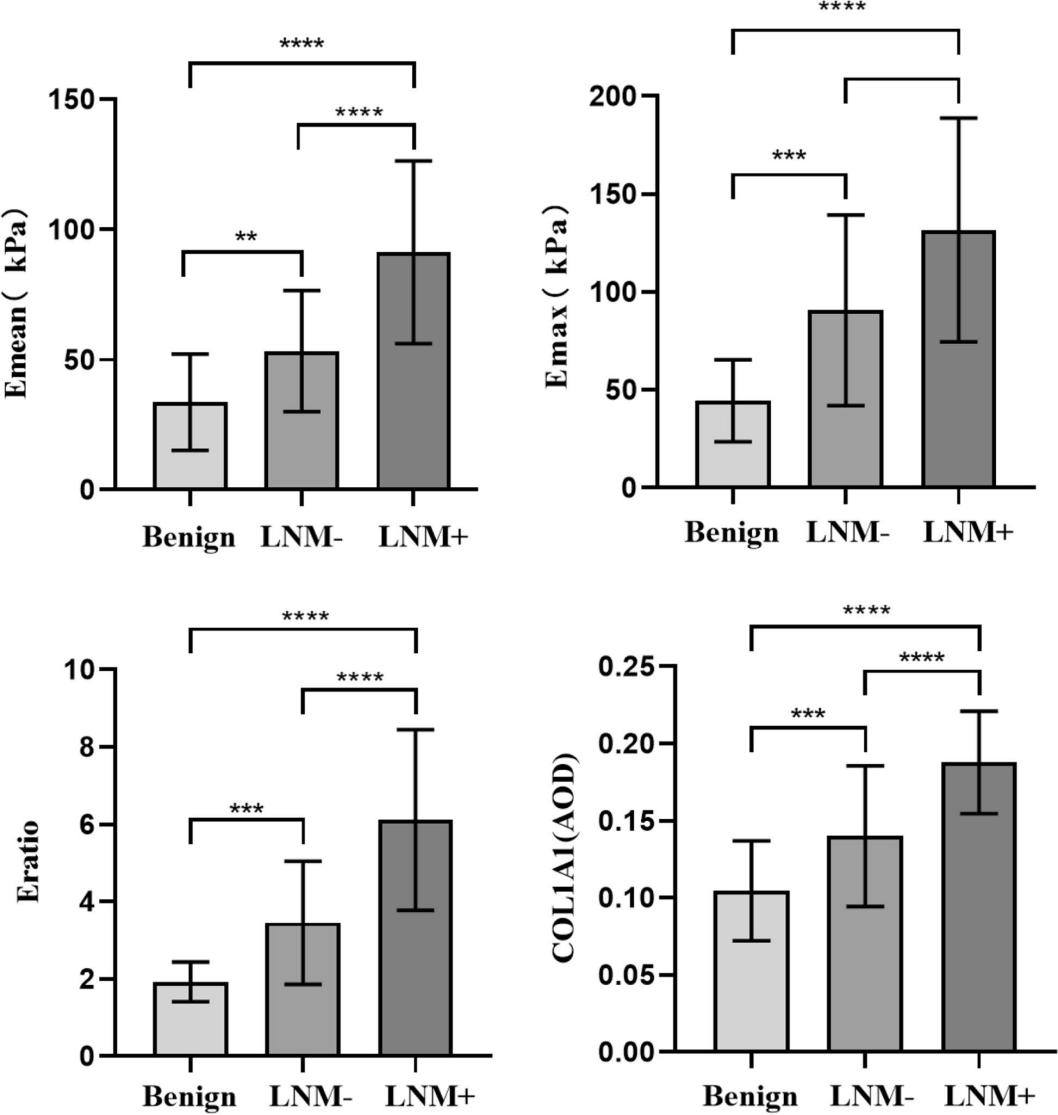
Pairwise comparison of elasticity parameters and COL1A1 expression levels in three groups. ****, *P* < 0.0001; ***, *P* < 0.001; **, *P* < 0.05; LNM, lymph node metastasis; COL1A1, collagen type I alpha 1; Emax, maximum elasticity; Emean, mean elasticity; Eratio, the elasticity ratio of the lesions to the peripheral tissue

The best cut-off values of SWE for diagnosing lymphatic metastasis were as follows: 107.5 kPa for Emax, 85.15 kPa for Emean, and 3.90 for Eratio. Eratio showed the best diagnostic performance according to the area under the curve (AUC), with a 95% confidence interval (CI) of 0.845 (0.754–0.937). A pairwise comparison of SWE parameters of the benign, LNM-, and LNM + groups showed that the difference was statistically significant (Fig. 4).

In this study, the collagen fiber shape and arrangement features in the breast lesions were classified into three categories. For all 94 lesions, one-way ANOVA showed that Emax, Emean, and Eratio differed between categories 0, 1, and 2 (all *P* < 0.05; Table 2). This shows that Emax, Emean, and Eratio increase when the collagen fiber class increases from 0 to 2. Furthermore, Kendall's tau-b correlation coefficient calculation showed that the correlation coefficient between Emax and collagen category was 0.318, that between Emean and collagen category was 0.261, and that between Eratio and collagen category was 0.349 (all *P* ≤ 0.001; Table 3).

**Table 2:** Elastic parameters between the different collagen fiber categories in 94 breast lesions

SWE values	Category of collagen fibers			*P* value
	**0**	**1**	**2**	
**Emax (kPa)**	64.670 ± 51.160	93.100 ± 54.654	112.010 ± 51.427	0.002
**Emean (kPa)**	47.280 ± 32.654	55.550 ± 31.357	73.440 ± 33.236	0.008
**Eratio**	2.710 ± 1.480	3.450 ± 1.617	5.340 ± 2.910	< 0.001

Emax, maximum elasticity; Emean, mean elasticity; Eratio, elasticity ratio of lesions to peripheral tissue

**Table 3:** Correlation between the different collagen fiber categories and SWE parameters in 94 breast lesions

	**N**	**Kendall’s tau-b correlation coefficient**	***P* value**
Emax vs. collagen fibers categories	94	0.318	< 0.001
Emean vs. collagen fibers categories	94	0.261	0.001
Eratio vs. collagen fibers categories	94	0.349	< 0.001

Emax, maximum elasticity; Emean, mean elasticity; Eratio, elasticity ratio of lesions to peripheral tissue

COL1A1 expression levels were used for differential diagnosis of benign and malignant breast lesions. The mean expression level of COL1A1 in benign breast lesions was 0.105 ± 0.323; in malignant breast lesions, the mean expression level was 0.159 ± 0.047 (*P* = 0.012). Furthermore, an average optical density > 0.145 was the cut-off value, the effective rate was 0.808 (0.723–0.894), and the sensitivity and specificity were 66% and 90%, respectively. When the expression level of COL1A1 was used to diagnose LNM- and LNM + malignant breast lesions, the average optical density > 0.150 was taken as the cut-off value, the effectiveness was 0.80 (0.69–0.91), and the sensitivity and specificity were 96% and 59%, respectively.

**Fig. 5:**
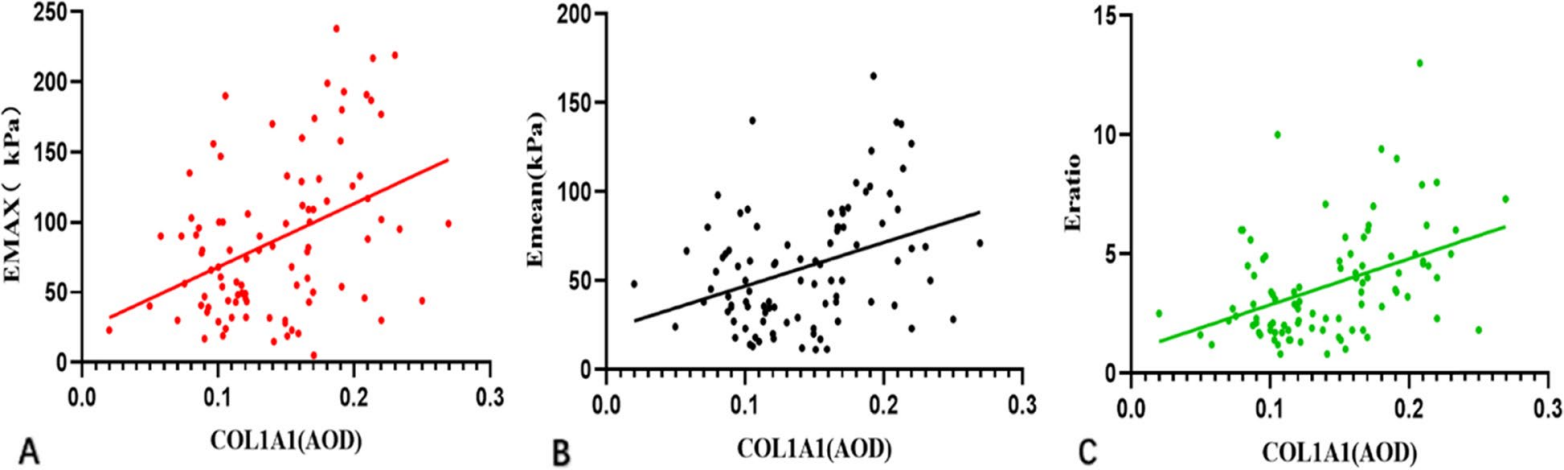
The expression level of COL1A1 was positively correlated with Emax(A), Emean(B), Eratio(C), (*r* = 0.406, 0.362, 0.425, respectively. *P* < 0.001). COL1A1, Collagen type I alpha 1; Emax, maximum elasticity; Emean, mean elasticity; Eratio, the elasticity ratio of the lesions to the peripheral tissue

A pairwise comparison of COL1A1 expression levels in the benign, LNM-, and LNM + groups showed that the difference was statistically significant (Fig. 4). Pearson correlation analysis showed a positive correlation between Emax, Emean, Eratio of breast lesions, and the expression level of COL1A1 (*r* = 0.406, 0.362, and 0.425, respectively). (*P* < 0.001; Fig. 5).

## Discussion

SWE is a new US diagnostic technology. It uses shear waves induced by acoustic radiation pulses to visualize and quantify tissue stiffness in a real-time, reliable, and reproducible manner [18]. Tissue stiffness is an important parameter for diagnosing potential malignancies or other diseases [19]. Our study showed that compared with benign breast lesions, malignant lesions had higher Emax, Emean, and Eratio, which was consistent with the results of several previous studies [20–22].

Furthermore, considering that preoperative knowledge of axillary lymph node status is crucial for the use of neoadjuvant chemotherapy and appropriate surgical treatment, it is necessary to develop imaging tools for LNM diagnosis [23, 24]. As expected from current trials, the main role of US on the breast in the near future may be to exclude the presence of axillary LNM [25, 26]. Our study showed that the best cut-off values of the SWE parameters for diagnosing lymphatic metastasis were calculated as follows: 107.5 kPa for Emax, 85.15 kPa for Emean, and 3.9 for Eratio.

According to ROC analysis, Eratio exhibited the best diagnostic performance with an AUC of 0.85 (0.75–0.94). Xin Wen et al. [27] reported that lesion stiffness was a predictor of axillary lymph node metastasis in breast cancer. Additionally, the optimal cut-off values of SWE parameters for predicting LNM were calculated as follows: 111.05 kPa for Emax, 79.80 kPa for Emean, and 6.89 for EmeanR. According to ROC analysis, Emax exhibited the best diagnostic performance with an AUC of 0.82 (0.76–0.87). This difference may have been caused by the following three factors: fewer cases were included in our study, the setting of the instrument and how Eratio was obtained (there is no relevant information in Wen's report), and operator-dependent changes.

Similarly, the present study demonstrated that the shape and arrangement of ECM collagen fibers are divided into three categories, which positively correlate with the Emax, Emean, and Eratio of breast lesion stiffness assessed by SWE. According to the results of all 94 lesions, Emax, Emean, and Eratio showed an increasing tendency from category 0 to category 2 (all *P* < 0.05).

In normal breast tissue, collagen fibers showed wavy or sinusoidal waves [13, 14]. Thus, this type of collagen fiber was assigned to category 0. Brabrand et al. reported that collagen in the peritumoral region was arranged in a more parallel alignment and aligned or linearized collagen correlated with increased stiffness of the breast tissue or lesions [10, 28]. We classified this type as class 1. Type 2 is characterized by bundles of straightened collagen fibers perpendicular to the tumor boundary, which can change into a honeycomb shape and show high hardness [16]. These results indicate that breast lesion stiffness increases when ECM collagen changes into taut, straightened and arranged parallel fibers. Additionally, a more complicated structure, collagen fibers connected in a honeycomb arrangement, indicated the highest stiffness. This indicates that ECM collagen fiber shape and arrangement may also contribute to the stiffness variance in malignant breast lesions.

The survival of patients with breast cancer is correlated with stromal biology, including the reorganization of the ECM to promote cancer invasion and migration [29]. Uddin et al. suggested that the expression of the stromal gene COL1A1 is associated with the progression of breast cancer and recurrence-free survival in breast cancer patients [30]. In our cohort, 30 tissue samples in the benign group and 64 tissue samples in the malignant group were consecutively included for COL1A1 immunohistochemical staining. Pearson correlation analysis showed a positive correlation between Emax, Emean, Eratio of breast lesions, and the expression level of COL1A1 (*r* = 0.406, 0.362, and 0.425, respectively; *P* < 0.001).

Furthermore, the average expression level of COL1A1 in benign breast lesions was significantly lower than that in malignant breast lesions. COL1A1 expression can be used to predict lymph node metastasis. An average optical density > 0.150 was taken as the cut-off value, the effectiveness was 0.80 (0.69–0.91), and the sensitivity and specificity were 96% and 59%, respectively. Moreover, many studies [31, 32] have also proved the correlation between collagen fiber content and SWE in breast lesions and proved that changes in collagen fiber content lead to changes in tumor tissue hardness. Li et al. [33] studied 148 triple-negative breast cancers and found that high COL1A1 expression in triple-negative breast cancer was an independent prognostic factor.

In addition, protein–protein interaction network analysis confirmed that COL1A1 is a prognostic matrix gene in breast cancer, and its expression is related to the progression of breast cancer [30]. These results suggest that COL1A1 may regulate tumor metastasis through some signaling pathways, and we have begun to investigate the possible signaling mechanisms using in vivo and in vitro methods.

This study had four limitations that need to be addressed. First, this study did not include the correlation between stiffness and other components of the ECM, such as elastic fibers, laminin, and fibronectin; however, collagen is the most important structural protein in the ECM, and its correlation with the elastic modulus may be the most important. Second, the number of included cases was small. Third, limited to the type of US application, examinations were performed by only one operator (no evaluation of inter-observer variability).

## Conclusions

The collagen fiber features and expression levels of COL1A1 were positively correlated with the elastic parameters of breast lesions. The expression of COL1A1 may be helpful in diagnosing benign and malignant breast lesions and predicting axillary lymph node metastasis.

## Data Availability

All data generated or analyzed during this study are included in this published article.

## Abbreviations

COL1A1: Collagen type I alpha 1
COL1A2: Collagen type I alpha 2
SWE: Shear wave elastography
US: Ultrasound
IBCs: Invasive breast cancers
ECM: Extracellular matrix
LNM: Lymph node metastasis
LNM -: Negative axillary lymph node metastasis
LNM +: Positive axillary lymph node metastasis
IOD: Integrated optical density
ROC: Receiver operating characteristic
AUC: Area under the curve
IHC: Immunohistochemistry
CI: Confidence interval
